# A case report of Stevens–Johnson syndrome caused by omeprazole

**DOI:** 10.1097/MD.0000000000039531

**Published:** 2024-09-06

**Authors:** Yuanhang Xu, Lingjuan Zhang, Lin Shen, Xueyan Guo

**Affiliations:** a Department of Gastroenterology, Shaanxi Provincial People’s Hospital, Xi’an, China.

**Keywords:** chronic and acute liver failure, omeprazole, Stevens–Johnson syndrome

## Abstract

**Rationale:**

Stevens–Johnson syndrome (SJS) is a rare but severe skin-mucosal reaction with a high mortality rate. It is characterized by sudden, painful blistering lesions on the skin, often accompanied by high fever and systemic toxicity. Lesions typically appear on the dorsal surfaces of the hands, feet, forearms, legs, and soles of the feet. They can also affect the conjunctiva, oral mucosa, labial mucosa, and vaginal mucosa. Patients may experience complications such as pneumonia, severe comorbidities, and liver and renal failure.

**Patient concerns:**

A 51-year-old female patient was admitted to the hospital due to “abdominal distention and skin yellowing for 20 days.” After using omeprazole, she developed a rash all over her body, and her liver function further deteriorated, ultimately leading to chronic acute liver failure.

**Diagnoses:**

The diagnosis included fever, rash suspected to be drug-induced, chronic and acute liver failure, and decompensation of post-Hepatitis B cirrhosis.

**Interventions:**

During hospitalization, suspected adverse drug reactions were discontinued, and symptomatic supportive treatment with methylprednisolone and fluid replacement was promptly provided.

**Outcomes:**

The patient’s symptoms and follow-up showed that the rash disappeared and liver and kidney function improved significantly after treatment.

**Lessons:**

We explored how chronic acute liver failure can cause immune system abnormalities and immune paralysis in patients, manifested as susceptibility to infection. This case report describes a drug-induced allergic reaction – SJS – in patients with chronic acute liver failure, as well as subsequent treatment, including hormone dosage and treatment duration. I hope this report will help enrich the relevant literature on drug-induced SJS combined with chronic and acute liver failure, laying the foundation for improving the survival rate of patients with the disease.

## 1. Introduction

Stevens–Johnson syndrome (SJS) is a rare but severe skin-mucosal reaction with a high mortality rate. It manifests as sudden, painful blistering lesions on the skin, often accompanied by high fever and systemic toxicity. These lesions typically appear on the dorsal surfaces of the hands, feet, forearms, legs, and soles. Additionally, involvement of the conjunctiva, oral mucosa, labial mucosa, and vaginal mucosa can occur. Patients with SJS are at risk of complications such as pneumonia, severe comorbidities, and liver and renal failure.^[1]^ This case report and analysis of omeprazole-induced SJS aims to increase clinical awareness of these side effects.

## 2. Case

The 51-year-old female patient was admitted to the hospital on February 11, 2022, complaining of abdominal distension and yellowing of the skin persisting for 20 days. Liver function tests revealed the following: 340 U/L for alanine aminotransferase, 728 U/L for aspartate aminotransferase, 61.3 g/L for serum total protein, 29.7 g/L for albumin, 227.8 μmol/L for total bilirubin, and 169.8 μmol/L for direct bilirubin. She was diagnosed with slow-onset acute liver failure and post-Hepatitis B cirrhosis upon admission. Her Hepatitis B viral DNA quantification (serum) was measured at 8.24 × 10^7^ IU/mL. Upon improvement in liver function and reduction in Hepatitis B DNA levels following bilirubin adsorption, hepatoprotective, and antiviral treatments, she was discharged on March 18, 2022. Medications prescribed included oral tenofovir disoproxil fumarate tablets, omeprazole enteric-coated capsules, and diammonium glycyrrhizinate enteric-coated capsules, which were continued postdischarge. On the day of discharge, she developed a rash at 5:00 pm, starting on the face and spreading sequentially to the upper limbs, palms, abdominal area, lower limbs, and soles of both feet. She also experienced fever, peaking at 39.2°C by 7:00 pm. A sore throat developed the following day, prompting her readmission to the hospital. The patient had no history of food or drug allergies.

**Figure 1.**
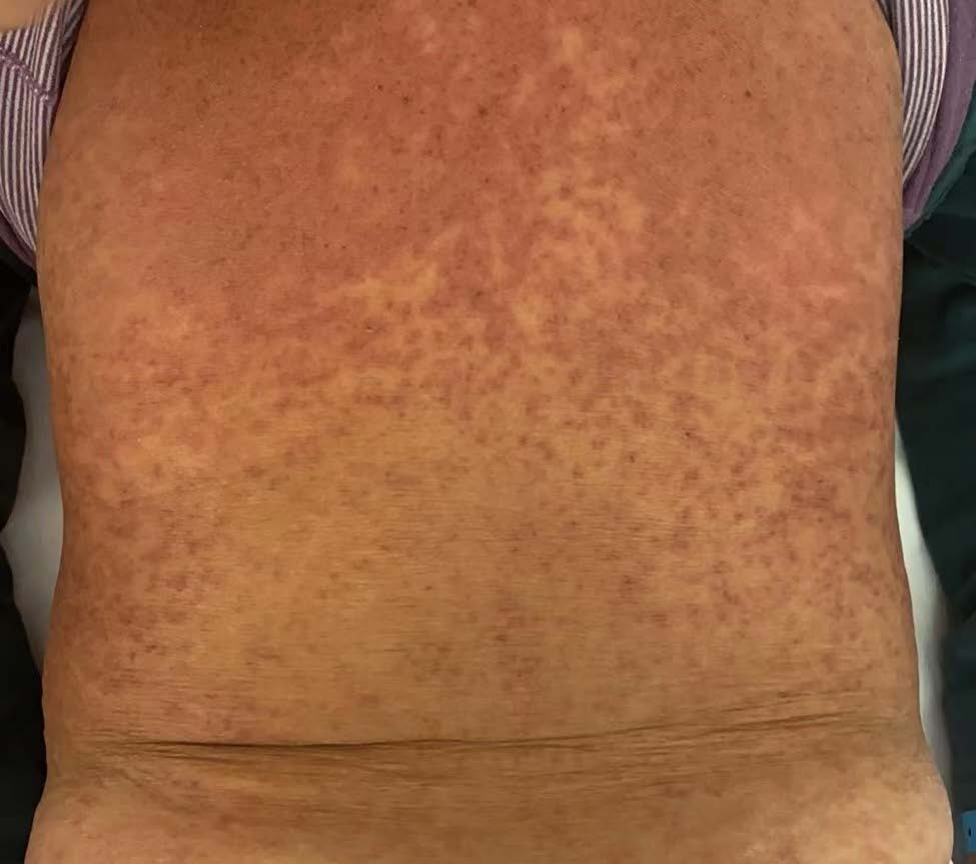
Red macules on the back.

**Figure 2.**
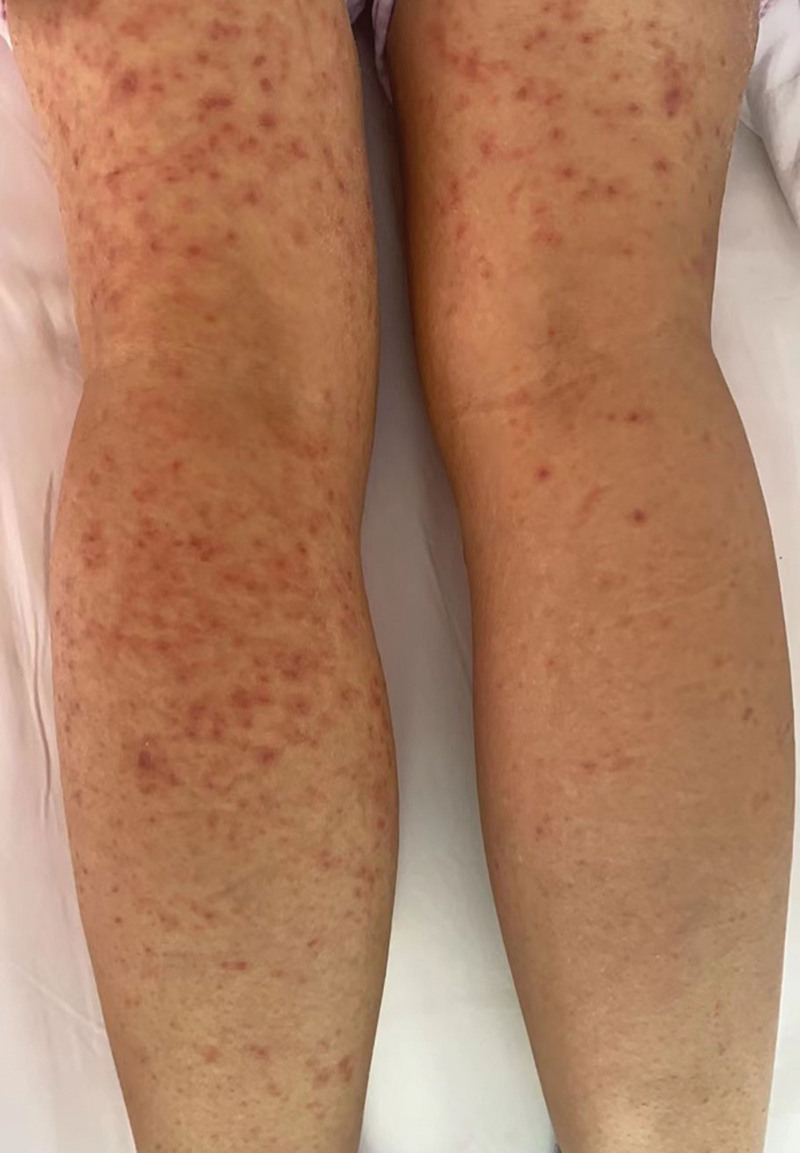
Red macules on both lower limbs.

Upon admission, the patient presented with a temperature of 39.2°C, pulse rate of 108 beats/min, respiratory rate of 19 breaths/min, and blood pressure of 108/59 mm Hg. Her height was 158 cm and weight was 65 kg. The patient exhibited moderate yellowish discoloration of the skin throughout the body. Diffuse erythema of varying sizes was observed on the skin surface, without any signs of hemorrhage (Figs. 1 and 2). No hemorrhages or petechiae were observed. The pharynx appeared slightly red, and bilateral tonsils were not enlarged. The heart rate was 108 beats per minute with arrhythmia, otherwise normal. Laboratory tests revealed a blood leukocyte count of 4.96 × 10^9^/L, alanine aminotransferase levels of 85 U/L, aspartate aminotransferase levels of 108 U/L, and serum albumin levels of 34.7 g/L (decreased). Admission diagnosis included fever and a rash warranting investigation, suspected to be drug-induced. The patient also presented with slow-accelerated hepatic failure and decompensated stage cirrhosis post-Hepatitis B. Clinical consultations from pharmacy, dermatology, and endocrinology specialists were sought. After ruling out nondrug-related causes, the patient was diagnosed with SJS, possibly induced by omeprazole or diammonium glycyrrhizinate. It was recommended to discontinue all medications except antiviral treatment.

**Figure 3.**
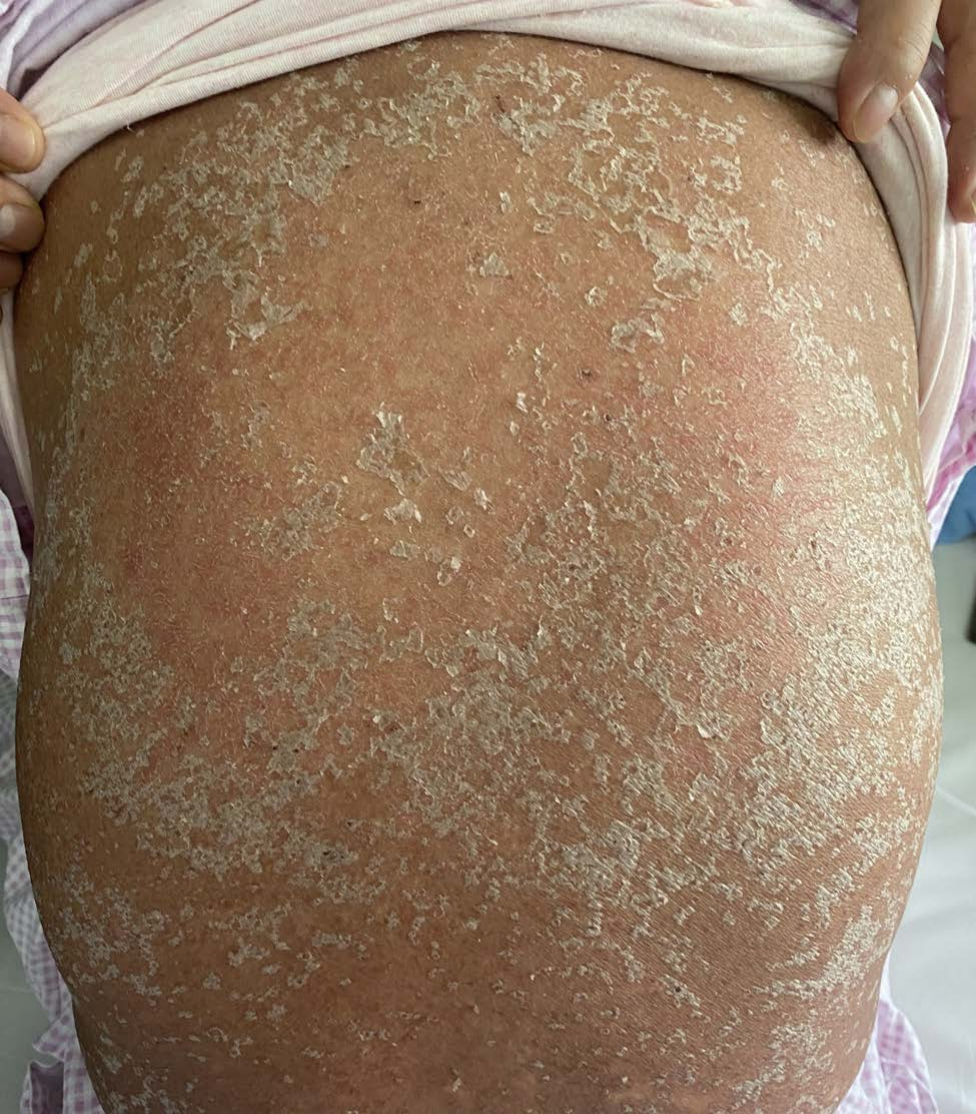
Recovery of the skin on the 10th day of hospitalization, with the rash beginning to crust and recede.

**Figure 4.**
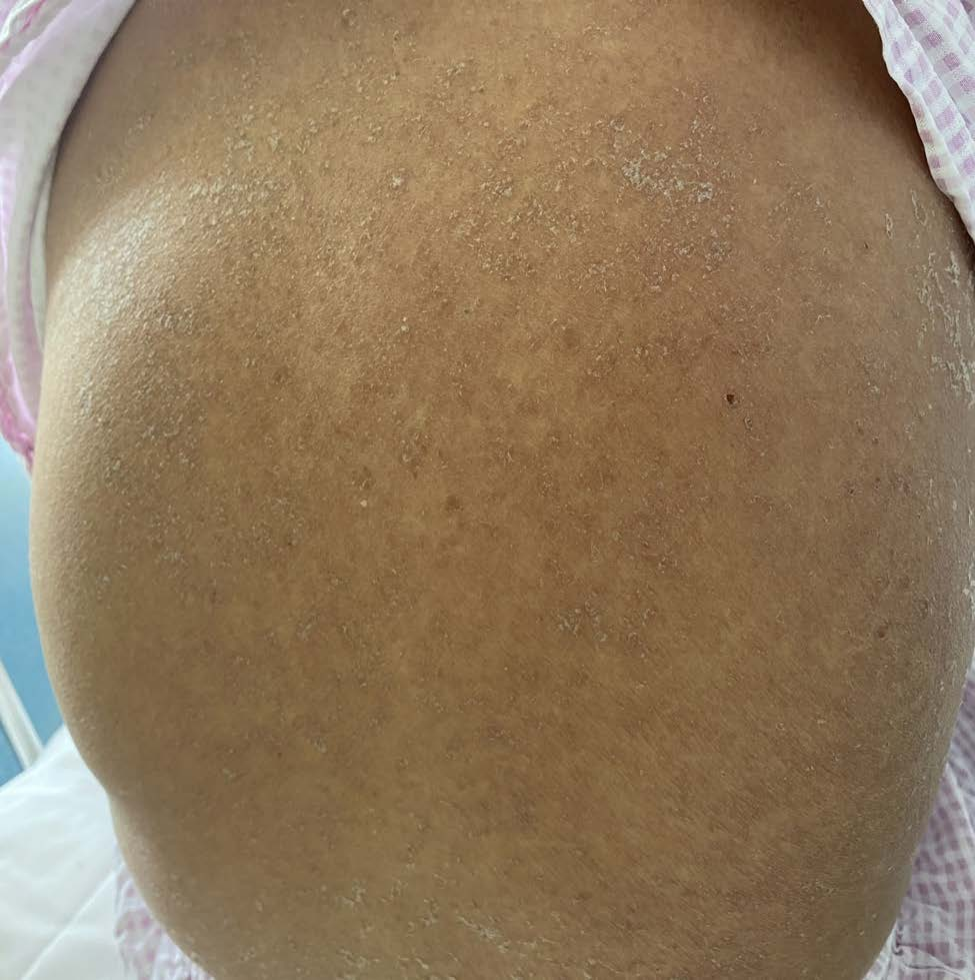
Recovery of the skin on day 18 of admission, with marked improvement of the rash and gradual healing of the lesion.

**Figure 5.**
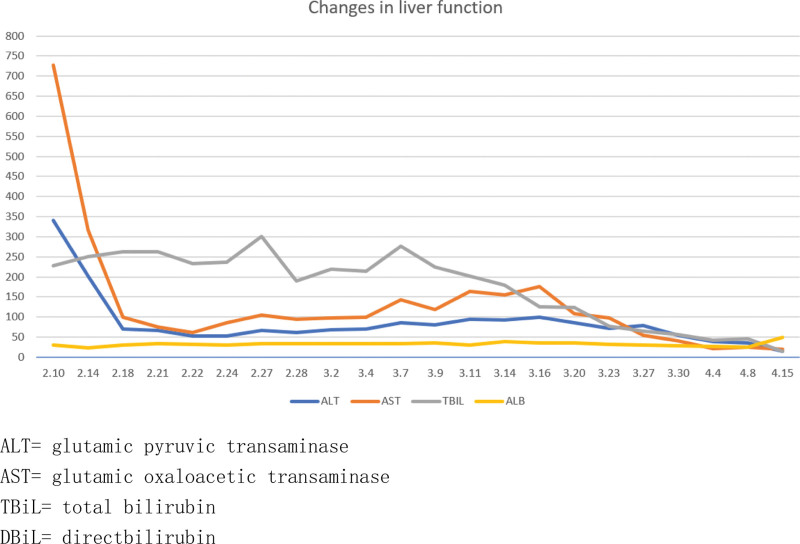
Changes in liver function.

**Figure 6.**
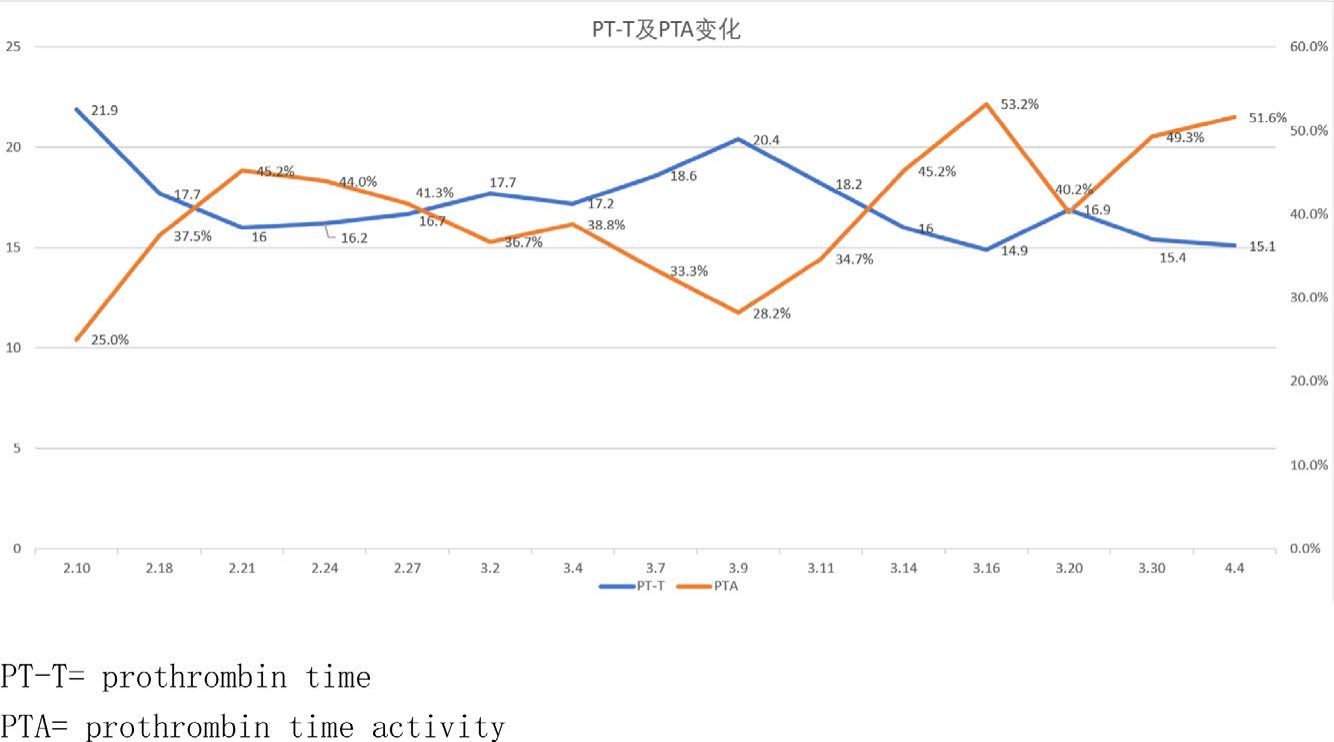
Changes in PT-T and PTA.

Omeprazole and diammonium glycyrrhizinate are the most likely causes of this syndrome and have been discontinued (Naranjo Adverse Reaction Probability Scale = 2), but the rash has not subsided. On day 5, the patient received intravenous methylprednisolone sodium succinate 40 mg/d, compound glycyrrhizin injection, and vitamin C injection for rehydration, along with cefotaxime sodium for infection prevention. On the 8th day, the erythema throughout the body did not spread further, and more rashes began to develop into dry scabs. There was improvement in the facial rash, prompting a reduction in the intravenous hormone dose to 20 mg/d. By the 10th day of admission, the generalized rash had developed dry scabs with no new eruptions. The rashes on the back and face started to crust and peel off (Fig. 3). Methylprednisolone tablets were adjusted to 16 mg in the morning and 12 mg in the afternoon. On the 14th day of admission, the patient showed no new rash or secondary infections, and relevant laboratory indicators such as leukocyte count and creatine kinase returned to normal. Consequently, the hormone dosage was reduced to 16 mg in the morning and 10 mg in the afternoon. On the 16th day of admission, the patient reported no significant discomfort. By this time, the entire body rash had peeled off and was recovering well, prompting a further reduction in hormone dosage to 16 mg in the morning and 8 mg in the afternoon. By the 18th day of admission, the patient’s skin rash had improved significantly with no new eruptions on the remaining parts of the body (Fig. 4). Review of relevant indicators showed decreased white blood cells with decreased neutrophils and an increased monocyte ratio. Bone marrow aspiration results indicated iron deficiency anemia and granulocytopenia. The patient’s condition was assessed as meeting discharge criteria. The dosage of methylprednisolone tablets was further reduced to 16 mg in the morning and 6 mg in the afternoon, with a planned reduction of 2 mg every 3 days. Electrolytes, blood glucose, liver function, and kidney function will be reassessed in the outpatient clinic after 1 week. The patient’s outpatient follow-up on April 15, 2022, showed satisfactory condition, with normal electrolytes, blood glucose, liver, and kidney function. Generalized hyperpigmentation and localized neoplastic epidermis were observed, with no blister formation. Specific changes in the patient’s liver function and prothrombin time and prothrombin time activity changes are illustrated in Figures 5 and 6.

## 3. Discussion

### 3.1. Omeprazole causes SJS-related reports

Physical examination is typically employed to diagnose SJS, while histological investigation confirms the diagnosis.^[2]^ Due to this, SJS was diagnosed despite incomplete skin histological examination, based on typical clinical signs such as fever and mucosal injury. The most common causes of SJS include anticonvulsants, antidepressants, sulfonamides, nonsteroidal anti-inflammatory drugs, antimicrobials, etc. Omeprazole is highly unlikely to be the cause of this condition.^[3]^ When discussing proton pump inhibitor medication guidelines, 1 of the extremely rare side effects associated with omeprazole enteric capsules is SJS. Seven cases of SJS induced by proton pump inhibitors were identified in PubMed when searching for proton pump inhibitors and SJS.^[4–10]^

### 3.2. Relevance evaluation

After reviewing the patient’s prescription history, examining pill inserts, and reviewing extensive literature, it was found that the patient may have developed SJS from taking omeprazole enteric capsules and diammonium glycyrrhizinate enteric capsules.

For individuals with SJS or toxic epidermal necrolysis (TEN) who have been exposed to multiple drugs, the ALDEN score is specifically designed to assess drug causality.^[11]^ The ALDEN scale was used to assess the 2 medications. Omeprazole enteric-coated capsules scored 4 (very likely), while diammonium glycyrrhizinate enteric-coated capsules scored −4 (very unlikely). Based on these results, omeprazole enteric-coated capsules are considered the drug most likely responsible for causing this patient’s SJS.

The specific scores are shown in Table 1. After further investigation, it was concluded that SJS is a delayed allergic reaction. The patient developed SJS 1 day after taking omeprazole, in a logical chronological sequence, and the probability of the reaction subsiding after discontinuing the medication was deemed very high. However, in our case, the patient was also diagnosed with hepatitis, and as liver function deteriorated further, he developed both chronic and acute liver failure. Patients with chronic acute liver failure typically exhibit compromised immune cell activity. Additionally, our patient showed a decrease in peripheral blood complement C3. Literature reports suggest that hepatitis infection accompanied by chronic and acute liver failure can predispose individuals to SJS/TEN.^[12]^ Based on current evidence, it cannot be denied that infection may also play a significant role in the development of SJS/TEN. However, the pathogenesis and specific mechanisms linking infection to SJS/TEN require further research. Given the above analysis, it is challenging to definitively determine whether omeprazole is indeed the primary cause of the disease onset, considering the Naranjo scale, hepatitis virus infection combined with chronic acute liver failure, and antibiotic treatment.^[13]^ SJS often leads to complications such as infection, acute respiratory distress syndrome, and shock from multiple organ failure. Therefore, a critical aspect of managing the disease’s progression is promptly identifying medications that may cause SJS through thorough history-taking and discontinuing them as soon as possible. Early detection and discontinuation of suspected sensitizing drugs, along with local care for damaged skin and mucous membranes, prompt initiation of appropriate anti-infective therapy, early fluid therapy, nutritional support, and timely administration of therapeutic drugs such as glucocorticoids, intravenous human immunoglobulin, and cyclosporine in critically ill patients, can significantly improve prognosis and reduce mortality rates.^[1]^ The mortality and prognosis of patients with SJS/TEN can be assessed using the SCORTEN scoring system.^[14]^ The SCORTEN score consists of 7 risk factors: age > 40 years; concomitant malignancy (including malignant solid tumors and malignant hematological disorders); heart rate > 120 beats/min; epidermal desquamation of >10% of the body surface area at the time of admission; (5) serum urea nitrogen level > 10 mmol/L; (6) serum glucose level > 14 mmol/L; serum bicarbonate level < 20 mmol/L. Our patient was admitted 1 day after onset of SJS with the following SCORTEN factors: age 51 years, >10% total body surface area involvement, serum bicarbonate level of 21 mmol/L, resulting in a SCORTEN score of 3 and a mortality rate of 32%. The score ranges from 0 to 7, corresponding to predicted risks of death of 1%, 4%, 12%, 32%, 62%, 85%, 95%, and 99%, respectively. In our case, the patient was initially diagnosed with chronic acute liver failure upon admission, and the rash did not initially present in a typical manner. As liver function and the rash worsened, managing the condition became increasingly challenging. The affected skin area expanded from <10% to involving most of the lower limbs and back, making it difficult to distinguish between SJS and TEN. In fact, it could more accurately be described as SJS/TEN overlap syndrome. Moreover, the patient’s compromised immune system increased the risk of developing sepsis. Therefore, supportive treatment focused on safety principles was provided. Unfortunately, the rash worsened on the 5th day of admission, prompting the initiation of intravenous methylprednisolone (40 mg, once daily). As the rash gradually improved, the dose of methylprednisolone was transitioned to oral administration and eventually discontinued altogether. Following treatment, the patient’s rash significantly subsided, blisters scabbed and peeled off, and the condition stabilized relatively.

**Table 1:** ALDEN scores.

Norm	ALDEN scoring rules	ALDEN rating	Omeprazole enteral	Diammonium glycyrrhizinate
Time elapsed between the date of initiation of drug use and the date of the clue	5 to 28 d	3	3	–
	29 to 56 d	2	–	–
	1 to 4 d	1	–	–
	>56 d	−1	–	–
	Drugs started on or after the day of the clue	−3	–	−3
	When an allergic reaction occurred in the same drug program in the past: 1 to 4 d (+3): 5 to 56 d (+1)			
Assess whether the drug is still present in the body at the time of the trial day	Continued use of the drug at the clue day or the time between the point of discontinuation of the drug and the clue day is less than 5 times the half-life of the drug	0	0	0
	The time between the point of discontinuation and the lead day is greater than 5 times the half-life of the drug, but the patient has abnormal hepatic/renal function or suspected drug interactions	−1	–	–
	The time between the point of discontinuation and the lead day is greater than 5 times the half-life of the drug, and the patient has no liver/renal abnormalities or drug interactions	−3	–	–
Re-introduction of the same drug/reaction to past use of the same ingredient or similar drugs	Re-introduction of the same drug causes SJS/TEN	4	–	–
	Re-administration of a similar drug causing SJS/TEN, or re-administration of the same drug causing other non-SIS/TEN allergic reactions	2	–	–
	Re-introduction of similar drugs causing other non-SIS/TEN allergic reactions	1	–	–
	Never used the same drug in the past	0	0	0
	Past use of the same or similar drugs without allergic reactions	−2	–	–
Continued use of the drug during the progression of SJS/TEN	Discontinued or unclear	0	0	0
	Continued use of the drug did not cause worsening of symptoms	−2	–	–
The drug’s degree of notoriety in history	High-risk drugs	3	–	–
	Drugs with identified but low risk	2	–	–
	Drugs under surveillance	1	1	–
	uncharted	0	–	0
	Drugs with no evidence of relevance	−1	–	–
There is more than 1 suspicious drug.	If there is more than 1 suspected drug, as long as the total score of the first 5 items of 1 of the drugs is more than plus or minus 3 points, all other suspected drugs will be deducted 1 point from the final total score.	−1	–	−1

Table 1 algorithm of drug causality for epidermal necrolysis scores.

Note: <0, very unlikely; 0 to 1, unlikely; 2 to 3, possible; 4 to 5, likely; ≥6, very likely.

SJS = Stevens–Johnson syndrome, TEN = toxic epidermal necrolysis, ALDEN = algorithm of drug causality for epidermal necrolysis.

To sum up, we present a case of SJS induced by omeprazole, complicated by both chronic and acute liver failure. Furthermore, we discuss the management of SJS concurrent with liver failure, focusing on corticosteroid dosing and duration of treatment. The potential occurrence of severe cutaneous adverse reactions underscores the importance for healthcare providers to remain vigilant, especially when treating patients with hepatitis viruses. SJS manifests as a severe systemic reaction primarily characterized by mucocutaneous damage, associated with high mortality rates. Early initiation of comprehensive systemic therapy is crucial to enhance patient outcomes and ensure medication safety.

## Author contributions

**Conceptualization:** Yuanhang Xu, Xueyan Guo.

**Data curation:** Yuanhang Xu, Lingjuan Zhang.

**Formal analysis:** Yuanhang Xu, Lin Shen.

**Investigation:** Yuanhang Xu, Xueyan Guo.

**Writing – original draft:** Yuanhang Xu.

**Writing – review & editing:** Lingjuan Zhang, Xueyan Guo.

## References

[1] Adverse Drug Reaction Research Centre, Chinese Medical Association, Dermatology and Venereology Branch. Expert consensus on the diagnosis and treatment of Stevens-Johnson syndrome/toxic epidermal necrolysis. Chin J Dermatol. 2021;54:376–81.

[2] Woolum JA Bailey AM Baum RA Metts EL. A review of the management of Stevens-Johnson syndrome and toxic epidermal necrolysis. Adv Emerg Nurs J. 2019;41:56–64. 30702535 10.1097/TME.0000000000000225

[3] Zhang L Li K Wu X. A case of Stevens-Johnson syndrome caused by esomeprazole. Zhongnan Pharmacol. 2023;21:3376–7.

[4] Daldoul M Charfi O Bouattour E. Pantoprazole-induced Stevens-Johnson syndrome: a case-report. Curr Drug Saf. 2024;19:148–50. 36823997 10.2174/1574886318666230224092818

[5] Lin YT Yang JC Chu CY. Esomeprazole-induced Stevens-Johnson syndrome in a patient who underwent nivolumab ab therapy for advanced lung adenocarcinoma. Lung Cancer. 2020;148:177–8. 32933772 10.1016/j.lungcan.2020.09.001

[6] Van Tendeloo E Gutermuth J Grosber M. Positive patch testing with omeprazole in Stevens-Johnson syndrome: a case report. J Eur Acad Dermatol Venereol. 2021;35:e74–5. 32654241 10.1111/jdv.16814

[7] Fracaroli TS Miranda LQ Sodré JL Chaves M Gripp A. Toxic epidermal necrolysis induced by lansoprazole. An Bras Dermatol. 2013;88:117–20. 23539016 10.1590/S0365-05962013000100018 PMC3699950

[8] Thakor AS Burke A Handfield-Jones S Sinha A Palmer M Burns A. Toxic epidermal necrolysis and neutropenia: complications of ome pyrazole. Australas J Dermatol. 2009;50:207–10. 19659985 10.1111/j.1440-0960.2009.00540.x

[9] Severino G Chillotti C De Lisa R Del Zompo M Ardau R. Adverse reactions during imatinib and lansoprazole treatment in gastrointestinal stromal tumors. Ann Pharmacother. 2005;39:162–4. 15546944 10.1345/aph.1E127

[10] Heaton NR Edmonds EV Francis ND Bunker CB Bowling JCR Morar N. Fatal toxic epidermal necrolysis due to lansoprazole. Clin Exp Dermatol. 2004;29:612–3. 15550134 10.1111/j.1365-2230.2004.01616.x

[11] Liu Y Wang W. Analysis of a case of Stevens-Johnson syndrome caused by capecitabine. Chin J Hosp Pharm. 2024;44:237–40.

[12] Zang X Chen S Zhang L Zhai Y. Toxic epidermal necrolysis in hepatitis A infection with acute-on-chronic liver failure: case report and literature review. Front Med (Lausanne). 2022;9:964062. 36213642 10.3389/fmed.2022.964062 PMC9537471

[13] Cokan A Gavrić Lovrec V Takač I. A case of Stevens-Johnson syndrome in recurrent late-stage ovarian cancer patient after management of chronic pain with elastomeric pump. Curr Oncol. 2021;28:2928–32. 34436022 10.3390/curroncol28040256 PMC8395433

[14] Bastuji-Garin S Fouchard N Bertocchi M Roujeau JC Revuz J Wolkenstein P. SCORTEN: a severity-of-illness score for toxic epidermal necrolysis. J Invest Dermatol. 2000;115:149–53. 10951229 10.1046/j.1523-1747.2000.00061.x

